# Multidisciplinary approach of early breast cancer: The biology applied to radiation oncology

**DOI:** 10.1186/1748-717X-5-2

**Published:** 2010-01-14

**Authors:** Céline Bourgier, Mahmut Ozsahin, David Azria

**Affiliations:** 1 Département d'Oncologie Radiothérapie, Unité fonctionnelle de Sénologie, Institut Gustave Roussy, Villejuif, France; 2 Service de Radio-Oncologie, Centre Hospitalier Universitaire Vaudois, Lausanne, Switzerland; 3 Département d'Oncologie Radiothérapie, Université Montpellier I, CRLC Val d'Aurelle, Montpellier, France

## Abstract

Early breast cancer treatment is based on a multimodality approach with the application of clinical and histological prognostic factors to determine locoregional and systemic treatments. The entire scientific community is strongly involved in the management of this disease: radiologists for screening and early diagnosis, gynecologists, surgical oncologists and radiation oncologists for locoregional treatment, pathologists and biologists for personalized characterization, genetic counselors for BRCA mutation history and medical oncologists for systemic therapies.

Recently, new biological tools have established various prognostic subsets of breast cancer and developed predictive markers for miscellaneous treatments.

The aim of this article is to highlight the contribution of biological tools in the locoregional management of early breast cancer.

## Introduction

Breast cancer is the most common female cancer in France with increasing incidence over the last two decades and with decreasing mortality [1]. Systematic screening can detects early breast cancers that are potentially curable. All physicians are implicated in the management of early breast cancer: radiologists for screening and diagnosis; gynecologists, breast surgeons, and radiation oncologists for locoregional treatment; pathologists and biologists for individualized tumor characterization; genetic counselors for BRCA mutation history and medical oncologists for systemic therapies.

Recently, biological tools have identified different prognostic subsets of breast cancers, and may predict treatment efficacy [2-8].

This review highlights the contribution of biological tools in a multidisciplinary approach, especially in locoregional treatment of early breast cancers.

## Biological tools

The advent of biological tumor analysis has generated information to classify prognostic features of breast cancer, and to assess treatment efficacy. Recently, several classifications were identified defining the genomic tumor characterization which include: (i) «intrinsic gene signature» [2,3], (ii) tumor proliferation or invasion [4-6], and (iii) tumor aggressiveness « wound signature; invasive gene signature » [7,8]. At least three molecular sets have therefore been suggested for breast cancer outcomes: luminal (positive hormonal receptors - HR), triple negative (negative HR/ negative Her2), and Her2 overexpression phenotypes [2-4]. Other biological tools have been developed to identify and classify those genes implied in tumor progression and treatment response, such as tissue micro arrays or comparative genomic hybridization (CGH)-arrays. Furthermore, these tools enable the detection of gene amplification or deletion that can be targeted by new systemic therapies [9].

## Differential diagnosis between benign and malignant breast lesions: contribution of biological tools

Recently, a new molecular classification based on differential expression of genes and exons has identified and generated a molecular classifier for breast cancer diagnosis through differentially expressed exons in malignant and benign breast lesions (accuracy 100% [IC95%: 96-100], sensitivity 100% [IC95%: 83-100%], and specificity 100% [IC95%: 95-100] [10]. This molecular signature for breast cancer diagnosis with fine-needle aspiration (FNA) is a promising biological tool owing to its safer and quicker procedure [10,11]. The FNA procedure has substantial advantages over core needle biopsy (CNB) even though the current guidelines recommend CNB for diagnosis of ACR grade 4 and 5 lesions and for assessment of tissue and molecular signature. First, FNA is a rapid and cost-effective tool for breast cancer diagnosis in a one-stop multidisciplinary breast clinic [12]. Moreover, FNA samples are more enriched in cancer cells than CNB samples and thus, provide transcriptional profiles of purer representation of the tumor-cell population [10,13,14]. In contrast, the main advantage of CNB compared to FNA is the ability to describe tumor architecture but it is more expensive and time-consuming [15].

## Breast cancer chemotherapy and the contribution of biological tools

The administration of systemic therapies is driven by the assessment of clinical and/or pathological features such as tumor size, nodal involvement, positive or negative hormonal receptors, and Her2 overexpression. However, none of these classical prognostic factors is able to predict the response to treatment of breast cancers. The advent of genomic technologies enables the improvement of prognostic classification and accurate prediction of benefit from systemic therapies for individual patients. The recent review of Di Leo et al. [16] analyzed and detailed all these biological tools and their clinical application, particularly in some ongoing clinical trials.

## The contribution of biology and endocrine therapies

Patients with hormonal receptor-positive breast cancers receive endocrine therapy given over five consecutive years, with either tamoxifen (TAM) in premenopausal women, or aromatase inhibitors (AI) or sequential endocrine therapy in postmenopausal women [17]. To determine which endocrine therapy would benefit a selected patient population is not feasible in daily practice. In this context, biological tools may identify patients in whom AI or TAM as initial endocrine therapy would be of benefit. Recently, Viale *et al*. have assessed the prognostic and predictive value of Ki-67 labeling index in the Breast International Group (BIG) trial 1-98. A high Ki-67 labeling index level was correlated to a worse disease-free survival (DFS), and patients treated by AI had an enhanced DFS compared to patients treated by TAM. The Ki-67 labeling index level could therefore identify a subgroup of patients who would benefit from initial AI endocrine therapy [18].

## Contribution of biology in adjuvant radiotherapy of early breast cancer

Biological technologies have been largely developed for classifying breast cancers after systemic therapies, and for predicting the efficacy of systemic therapies, whereas biological contribution in the field of locoregional treatment is less developed. Biological applications are warranted to optimize local control for breast cancer patients at high risk of local relapse. Furthermore, these biological tools would identify patients at high risk of late radiation-induced toxicity.

### Biological tools: To determine breast cancer patients at high risk of local relapse

The current locoregional management of early breast cancer consists of breast conserving surgery (i.e., lumpectomy and sentinel node biopsy or axillary dissection) followed by whole-breast irradiation (WBI, 50 Gy in 25 fractions over 5 weeks) and then a boost of 10-16 Gy to the tumor bed. WBI significantly reduces local relapse by a factor of two with an absolute gain in local relapse rate of 20% at 15 years, corresponding to a survival benefit of 5% [19]. The boost to the tumor bed contributes to a decrease in the local relapse risk of 50% whatever the patients' age [20-22].

While adjuvant radiotherapy plays a crucial part in the locoregional management of early breast cancer, local relapse still occurs. These local relapses are an independent factor for distant metastatic relapse [23] and for specific cancer mortality [24]. A number of clinical and histological parameters are usually described as prognostic factors for locoregional relapse such as young age, positive or close margins, extensive DCIS [25-28], high tumor grade, presence of vascular embolus and/or lymphovascular invasion (LVI), and tumor size [29-31]. However, none of these parameters are able to predict which patients are at high risk of locoregional relapse. Recently, a computer-based tool "IBTR !" attempted to predict local relapse in women with invasive breast cancer after breast conserving surgery. This nomogram is only valid for patients presenting low risk of local recurrence [32,33]. As clinical, histological, and computer-based tools are not sufficient to predict local relapse, prognostic and predictive biological factors are needed. A tissue-micro-array has been built, based on an "intrinsic gene signature" showing that Her2 overexpression and basal-like breast cancer subtypes were prognostic factors for local relapse [34]. Other genomic classifications could be used to determine the risk of local relapse after breast conserving surgery, such as "wound signature" [35].

A history of BRCA1/2 mutation is related to a higher lifetime risk of developing breast cancer and breast conserving treatment remains debatable in this patient population owing to the residual presence of breast tissue which still contains all remaining cells carrying the same deleterious mutations. The BRCA1/2 mutation is not yet a targeted tool to determine a population of breast cancer at high risk of local relapse. The molecular pathway involved in DNA repair, particularly the role of BRCA1/2 proteins, would suggest a profile of tumor resistance to ionizing radiation (IR) in case of BRCA1/2 mutation, due to underlying *in vitro *effects. An incorrect repair of DNA double-strand breaks after IR, due to BRCA1/2 mutation, could lead to the development of new primary breast cancer. On the other hand, the *in vitro *effects of IR on BRCA1/2 cell lines showed an increased radiosensitivity [36,37], partially related to the loss of bcl-2 expression after IR, leading to an increased radiation-induced apoptosis [38]. Clinical data suggest that BRCA1/2 carriers have a similar local and overall outcome to non-carriers whatever radical or conservative local treatment has been performed [39,40].

### Biological tools: a need for combined therapies

Breast cancer cell lines that overexpressed Her2 oncoprotein are known to be resistant to IR. When trastuzumab was combined with IR, an enhancement of *in vitro *and *in vivo *tumor radiosensitization was observed, through DNA repair inhibition and through an increased tumor cell death [41,42]. Recently, the addition of trastuzumab to radiotherapy has been shown to be an effective radiosensitizer in patients with Her2 overexpression, with chemotherapy-refractory disease, locally-advanced or recurrent breast cancer in a phase II trial. Although these breast cancers were initially considered to be inoperable, breast surgery could be performed in 58% of patients with a substantial pathologic response (complete response or microscopic residual disease) in 43% of patients [43].

### Biological tools: To a personalized radiosensitivity of normal tissues

Different parameters could be involved in the development of late radiation-induced toxicity, such as genetic factors (DNA repair deficiency) or epigenetic factors (obesity, vascular, or collagen diseases...). Other issues could also be involved in radiation-induced toxicities such as radiotherapy parameters (total dose, dose per fraction, irradiated target and normal tissue volumes), history of surgery within irradiated fields, and the combination of either chemotherapy or endocrine therapy with radiation therapy.

Recently, a nomogram was built to predict the risk of fibrosis after breast-conserving therapy [44]. This computer-based tool allocated points according to various factors such as age, postoperative hematoma, breast edema, the use (concomitant or not) of tamoxifen and/or chemotherapy, radiotherapy parameters (photon energy [6 MV or more], electron or photon boost, energy of electron boost in case of electron use, maximal total dose). The main limitation for daily use of this nomogram is that this computer-based tool cannot identify a population at high risk of severe late radiation-induced toxicities. Indeed, when usual radiotherapy parameters were considered, i.e., 6-MV photons, a total dose of 66 Gy and photon beam boost, the nomogram predicts an over-risk of fibrosis of 50% at 10 years. This over-risk is larger than observed fibrosis after breast conserving therapy.

Biological predictive factors are warranted to identify the individual risk of development of severe late toxicity. A lymphocyte apoptosis assay has been developed as a rapid tool for characterization of normal tissue radiosensitivity, particularly due to the ease of blood collection in a standardized, patient-friendly manner [45-47]. Severe late radiation-induced toxicities (grade 2 or more) were correlated to a low rate of radiation-induced CD8 T-lymphocyte apoptosis (≤ 16%) [48,49]. In addition, patients with severe late effects possessed four or more SNPs (Single Nucleotide Polymorphisms) in candidate genes (ATM, TGFB1, XRCC1, XRCC3, SOD2, and RAD21) [48].

## Conclusion

Biological tools can play a part in each step of the management of early breast cancer from diagnosis to treatment; i.e., surgery, adjuvant radiotherapy, and systemic therapies. Currently used biological tools for prognostic classification or for predicting systemic treatment efficacy, could be applied for locoregional treatments to predict antitumor efficacy. In addition, radiation oncologists have developed new tools focusing on normal tissue radiosensitivity that may be adapted to systemic therapies in the near future. Dedicated prospective studies are urgently warranted in this setting.

## Conflict of interests

The authors declare that they have no competing interests.

## Authors' contributions

CB, MH and DA: conception, design. All the listed authors have been involved in drafting or in revising the manuscript. All authors read and approved the final manuscript.
